# Characterization of a novel secretory spherical body protein in *Babesia orientalis* and *Babesia orientalis*-infected erythrocytes

**DOI:** 10.1186/s13071-018-3018-y

**Published:** 2018-07-25

**Authors:** Jiaying Guo, Muxiao Li, Yali Sun, Long Yu, Pei He, Zheng Nie, Xueyan Zhan, Yangnan Zhao, Xiaoying Luo, Sen Wang, Siqi Aoyang, Qin Liu, Cuiqin Huang, Lan He, Junlong Zhao

**Affiliations:** 1 State Key Laboratory of Agricultural Microbiology, College of Veterinary Medicine, Huazhong Agricultural University, Wuhan, 430070 Hubei China; 2 Key Laboratory of Animal Epidemical Disease and Infectious Zoonoses, Ministry of Agriculture, Huazhong Agricultural University, Wuhan, 430070 Hubei China; 3 Key Laboratory of Preventive Veterinary Medicine in Hubei Province, Wuhan, 430070 Hubei China; 4 College of Life Science, Longyan University & Fujian, Provincial Key Laboratory for the Prevention and Control of Animal Infectious Diseases and Biotechnology, Longyan, 364012 Fujian China

**Keywords:** Spherical body, Apical organelle complex, *Babesia orientalis*, Native form, Immunoreactivity, Cellular localization

## Abstract

**Background:**

The spherical body, a membrane bound organelle localized in the apical organelle complex, is unique to *Babesia* and *Theileria* spp. The spherical body proteins (SBPs) secreted by spherical bodies include SBP1, SBP2, SBP3 and SBP4. Up to now, only SBP3 has been characterized in *Babesia orientalis*.

**Methods:**

The BoSBP4 gene was amplified from cDNA and gDNA and cloned into the pGEX-6P-1 vector by homologous recombination, sequenced and analyzed by bioinformatics tools. The amino acid (aa) sequence of BoSBP4 was compared with that of *Babesia bovis* and *Babesia bigemina* as well as SBP3 of *B. orientalis*. The immunoreactivity was evaluated by incubating recombinant BoSBP4 (rBoSBP4) with the serum of *B. orientalis*-infected water buffalo. The native form of BoSBP4 was identified by incubating lysate of *B. orientalis*-infected water buffalo erythrocytes with the anti-rBoSBP4 mouse serum. The cellular localization of BoSBP4 was determined by indirect immunofluorescence assay.

**Results:**

The full length of the BoSBP4 gene was estimated to be 945 bp without introns, encoding a 314 aa polypeptide with a predicted molecular weight of 37 kDa. The truncated recombinant protein was expressed from 70 to 945 bp as a GST fusion protein with a practical molecular weight of 70 kDa. BoSBP4 shared a 40% and 30% identity with *B. bovis* and *B. bigemina*, respectively. Furthermore, it was 31% identical to SBP3 of *B. orientalis*. BoSBP4 was identified in the lysate of *B. orientalis*-infected water buffalo erythrocytes with a molecular weight of 37 kDa, corresponding to the expected molecular mass of BoSBP4. The result of rBoSBP4 with positive serum revealed that BoSBP4 can elicit an immune response to *B. orientalis-*infected water buffalo. The cellular localization of BoSBP4 was detected to be adjacent to the merozoite nucleus in the intracellular phase, followed by the diffusion of the fluorescence of BoSBP4 into the cytoplasm of *B. orientalis-*infected erythrocytes as puncta-like specks and a gradual increase of the fluorescence.

**Conclusions:**

In this study, SBP4 in *B. orientalis* was characterized for the first time. It may play a key role in interaction with the host cell by being secreted into the cytoplasm of the *B. orientalis-*infected erythrocytes to facilitate parasite growth and reproduction.

## Background

Babesiosis is caused by species of the genus *Babesia*, and is one of the most epidemic infections distributed throughout the world [1–3]. *Babesia* spp. can cause different kinds of babesiosis in animals, and buffalo babesiosis greatly affects the cattle industry, leading to huge economic losses annually [3, 4]. It has been reported that several parasites can cause buffalo babesiosis, including *Babesia bovis*, *Babesia bigemina* and *Babesia orientalis* [4, 5]. *Babesia orientalis* was identified to be transmitted by *Rhipicephalus haemaphysaloides* in its original description in 1997 [6, 7]. Clinical symptoms for *B. orientalis* infection include anemia, fever, icterus and hemoglobinuria, and it can cause death in serious cases [3, 8].

To successfully invade the host cell, protozoan parasites need to depend on the parasite-derived proteins secreted by apical complex organelles [9, 10]. The apical complex organelles consist of rhoptries, micronemes and dense granules [10]. Previous studies have shown that the proteins discharged by rhoptries and micronemes mainly participate in the initial attachment, invasion and the early stage of post-invasion processes [11–14]. Dense granules are reported to release proteins into the parasitophorous vacuole (PV) shortly after invasion, which may play a key role in PV membrane modifications and is assumed to be associated with the nutrient acquisition. Likewise, the proteins secreted by dense granules are also translocated to the cytoplasmic side of infected red blood cell (iRBC) and participate in stabilizing spectrin tetramers [15–18]. However, in *Babesia* spp. and *Theileria* spp., spherical bodies are membrane-bound and localized to the apical organelles complex instead of dense granules [9, 19]. The spherical body proteins (SBPs) secreted by spherical bodies are identified to belong to a family consisting of SBP1, SBP2, SBP3 and SBP4, which have been characterized in *B. bovis* [20–22]. Using immunoscreening, SBP1 was firstly identified from the genomic DNA (gDNA) of *B. bovis* merozoite with a molecular weight of 77 kDa and located at the apex of the intraerythrocytic parasite [23]. In 1995, SBP1 was characterized to be localized to the spherical bodies by immunofluorescence and immunoelectron microscopy [19]. For SBP2, previous studies have reported a 225 kDa protein from *B. bovis*, which is localized to the cytoplasmic side of the iRBC [22, 24]. SBP3 and SBP4 of *B. bovis* have been recently characterized and identified to localize to the cytoplasm of iRBC, rather than the cytoplasmic side of the iRBC [20, 21, 25]. However, only the SBP3 of *B. orientalis* has been characterized so far in the merozoite to be discharged to the cytoplasm of iRBC [26].

Despite extensive research on SBPs and the identification of SBPs many years ago, their mechanisms and functions remain poorly understood. Furthermore, many studies have shown that antibodies against SBPs play significant roles in the protection of *Babesia*-infected cattle and are potential candidates for the development of vaccines [27–29]. Therefore, it is necessary to further investigate SBPs by evaluating their functions in interaction with the host cells.

## Methods

### Identification, cloning and sequencing of the BoSBP4 gene

In order to find the BoSBP4 gene, the genome of *B. orientalis* (unpublished data) was screened using a Basic Local Alignment Search Tool (BLAST) (https://blast.ncbi.nlm.nih.gov/Blast.cgi), TBLASTN, with the reported SBP4 amino acid sequence of *B. bovis* (GenBank: AAL92106) and *B. bigemina* (GenBank: XP_012767973) as queries [3, 30, 31]. The gene with a significant similarity with SBP4 of *B. bovis* and *B. bigemina* was designated as BoSBP4 gene.

The following primers for cloning BoSBP4 gene were designed based on the *in silico* BLAST search result and the *B. orientalis* genome sequence: the forward primer (5'-ATG GTG GCT CTT TCC CTA CG-3') and the reverse primer (5'-TTA CTC AGT GGT GGT TTC GGT TTC-3').

The recombinant plasmid was constructed using the homologous recombination method. The following primers for cloning BoSBP4 gene and the pGEX-6p-1 vector were synthesized (Tianyi Huiyuan Biological Technology, Wuhan, China): the forward primer for cloning BoSBP4 gene (5'-TTC TGT TCC AGG GGC CCC TGG AGG AAG TTG TTG AGG AAC C-3') and the reverse primer (5'-GAT CGT CAG TCA GTC ACG AT GTT ACT CAG TGG TGG TTT CGG-3'); the forward primer for cloning pGEX-6p-1 vector (5'-CAT CGT GAC TGA CTG ACG ATC-3') and the reverse primer (5'-CAG GGG CCC CTG GAA CAG AA-3').

The complementary DNA (cDNA) and gDNA of *B. orientalis* were extracted and stored at -80 °C as reported previously [26]. The entire length of the BoSBP4 gene was cloned from cDNA and gDNA. For construction of the recombination plasmid, the BoSBP4 gene and vector were amplified respectively from gDNA of *B. orientalis* and pGEX-6p-1 plasmid (Takara Biotechnology, Beijing, China) using corresponding homologous recombination primers as described above. The thermal cycling parameters for BoSBP4 gene included the initial denaturation at 95 °C for 5 min; 33 cycles of denaturation at 94 °C for 30 s, annealing at 55 °C for 30 s, extension at 72 °C for 3 min; and a final extension of 10 min at 72 °C. The vector cloning included the initial denaturation at 95 °C for 5 min; 33 cycles of denaturation at 94 °C for 30 s, annealing at 55 °C for 30 s, extension at 72 °C for 6 min; and a final extension of 10 min at 72 °C.

The PCR products were electrophoresed using 2% ethidium bromide-stained agarose gel and purified using an EasyPure® Quick Gel Extraction Kit (Invitrogen, Carlsbad, CA, USA). The purified BoSBP4 gene was ligated into extraction product of pGEX-6p-1 vector using a ClonExpress II One Step Cloning Kit (Vazyme Biotech, Nanjing, China). The pGEX-6p-1 vector sequencing primers were used for sequencing the BoSBP4 gene.

### Phylogenetic and bioinformatics analysis

The amino acid sequence of BoSBP4 was aligned with homologous sequences using MAFFT version 7 (https://mafft.cbrc.jp/alignment/server/) [32, 33]. Phylogenetic analysis was performed by the maximum likelihood method using MEGA 6.0 software [34].

The numbers of introns and exons were analyzed with the GENSCAN online tool (http://genes.mit.edu/GENSCAN.html) [35]. The predicted amino acid sequence of BoSBP4 was obtained using ExPASY online tool (http://www.expasy.org/translate/. Open reading frame (ORF) was analyzed by ORF finder (https://www.ncbi.nlm.nih.gov/orffinder/) [36]. Transmembrane (TM) regions, putative glycosylphosphatidy-l-inositol (GPI) anchors and putative signal peptides were predicted by TMHMM 2.0 (http://www.cbs.dtu.dk/services/TMHMM-2.0/), GPI Prediction Server 3.0 (http://mendel.imp.ac.at/sat/gpi/gpi_server.html) and SignalP 4.1 Server (http://www.cds.dtu.dk/dervices/signalp/), respectively [37, 38]. The potential subsequence motifs were analyzed with Motif Scan Server (http://mythis.isb-sib.ch/cgi-bin/motif_scan).

The secondary structure of BoSBP4 was predicted using the PROTEAN program of the Lasergene® software package in DNAstar in terms of hydrophilicity, flexible region, antigenic index and surface probability. For the prediction of tertiary structure, the amino acid of BoSBP4 was submitted to the I-TASSER server (https://zhanglab.ccmb.med.umich.edu/) [39–41]. There were approximately three steps for prediction. First, structure templates were identified from the PDB library using the local meta threading server (LOMETS) by accumulating high scoring targets for template alignments from nine threading programs [40, 42]. Ten top threading templates were selected due to higher structure accuracy in conserved regions with the amino acid of BoSBP4. Secondly, based on the above threading template alignments and the five largest structure clusters using SPICKER program, five models were selected [39, 41]. The one with the best quality among the five selected models was considered as the preliminary tertiary structure of the BoSBP4. Finally, the above selected model was matched with all the structures of proteins in the PDB library and ten top proteins were collected due to their closest structural similarity.

### Expression and purification of the recombinant protein

Because there was a predicted cleavage site between 22 and 23 aa based on the SignalP 4.1 Server prediction website (http://www.cds.dtu.dk/dervices/signalp/), the BoSBP4 gene was truncated and expressed from 70 to 945 bp for the convenience of prokaryotic expression.

The pGEX-6P-1/BoSBP4 truncated recombinant plasmid was confirmed by restriction enzyme digestion and sequencing analysis, then was transformed into *E. coli* BL21 (DE3) strain. Small-scale culture was subjected to 1 mM isopropyl-β-D-thiogalactopyranoside (IPTG) induction at 37 °C for 4 h. The expression form was analyzed by sodium dodecyl sulfate polyacrylamide gel electrophoresis (SDS-PAGE). The recombinant BoSBP4 (rBoSBP4) was expressed as GST-fusion protein and purified using Glutathione Sepharose HP (GE Healthcare, Pittsburgh, PA, USA). In brief, the *E. coli* cells were washed three times with phosphate buffer saline (PBS), and lysed by ultrasonication in binding buffer containing phenylmethylsulfonyl fluoride (PMSF). After centrifugation at 12,000× *g* for 15 min at 4 °C, the supernatant was collected, purified with the Glutathione Sepharose HP (GE Healthcare) and stored at -80 °C for further use.

### Polyclonal antibody preparation

The rBoSBP4 (100 μg each) in Freund’s complete adjuvant (Sigma, San Francisco, CA, USA) was subcutaneously injected into five mice (specific pathogen free). The same antigen administration in Freund’s incomplete adjuvant (Sigma) was conducted on the 14, 21 and 28th day. Serum from the five immune mice was collected 14 days after the last immunization and the serum from the healthy mice was collected as control and stored at -20 °C. The antibody titers of the immune serum were determined by the enzyme-linked immunosorbent assay (ELISA).

### SDS-PAGE and Western blot

To evaluate if *B. orientalis-*infected water buffalo can generate antibodies against SBP4, rBoSBP4 was verified by SDS-PAGE and subsequently blotted on a polyvinylidene fluoride membrane (PVDF, Merck Millipore, Kenny, NJ, USA). The membrane was blocked with 0.05% Tween-20 in tris buffered saline (TBST) plus 5% skimmed milk and then probed separately with the serum from *B. orientalis* infected water buffalo (1:400) or normal water buffalo. A secondary antibody (1:2000) of horseradish peroxidase (HRP)-conjugated anti-bovine IgG (Bioss, Beijing, China) was used to identify the bound proteins on the blots.

To identify the native form of SBP4 in *B. orientalis* merozoite, lysates of *B. orientalis*-infected water buffalo erythrocytes were incubated with the anti-rBoSBP4 mouse serum. Briefly, lysates of *B. orientalis-*infected and uninfected water buffalo erythrocytes were separated on 12% SDS–PAGE gel, blotted onto a PVDF (Merck Millipore, Kenny, NJ, USA) and probed with the anti-rBoSBP4 mouse serum (1:400) or the serum of naïve mouse or anti-GST tag mouse monoclonal antibody (1:1000, Proteintech Group, Chicago, USA) as controls. The membranes were washed with TBST and then incubated with goat anti-mouse IgG/HRP (1:2000) as secondary antibodies (Bioss). Protein bands on membranes were visualized using the DAB method (ZSGB-BIO, Beijing, China).

### Indirect immunofluorescence assay

Indirect immunofluorescence assay (IFA) was carried out to determine the cellular localization of BoSBP4. In brief, *B. orientalis*-infected and normal water buffalo erythrocyte smears were fixed in cold 50% acetone: 50% methanol (v:v) solution for 30 min at -20 °C and then incubated with the anti-rBoSBP4 mouse serum (1:200), the naïve mouse serum (1:200, negative control) or anti-GST tag mouse monoclonal antibody (1:1000, negative control, Proteintech Group) for 1 h at 37 °C. After washing three times with the cold PBS, smears were incubated with the second antibody of Alexa-Fluor® 594-conjugated goat anti-mouse IgG (1:1000 dilution; Invitrogen) for 1 h at 37 °C. For nuclear staining, smears were incubated with Hoechst (1:1000 dilution, Invitrogen). Another negative control consisted of the second antibody only without incubation with the primary antibody. Imaging was performed using fluorescence microscopy.

## Results

### Characterization of BoSBP4

**Fig. 1:**
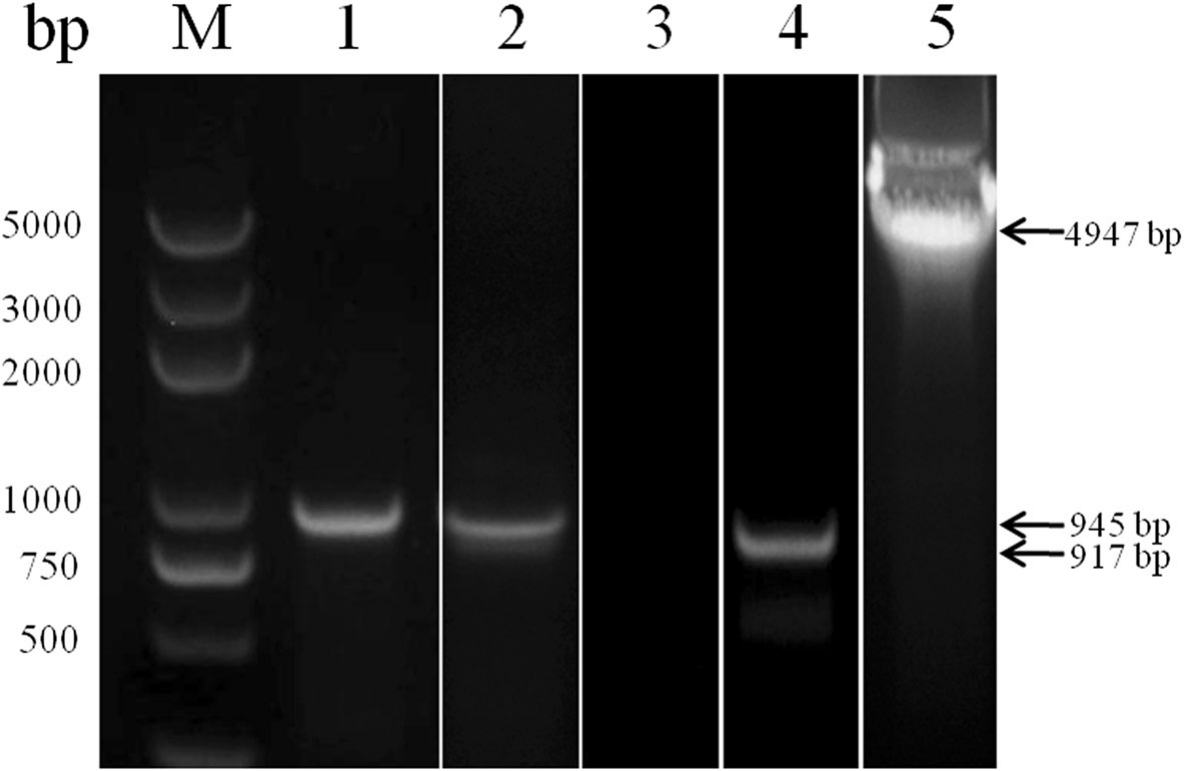
PCR amplification of the BoSBP4 gene from *B. orientalis* gDNA and linear pGEX-6P-1 fragment from pGEX-6P-1 plasmid. Lane M: marker; Lane 1: amplificon from gDNA; Lane 2: negative control; Lane 3: amplificon from gDNA for recombinant plasmid construction; Lane 4: amplificon from plasmid of pGEX-6P-1 for recombinant plasmid construction. The corresponding bands are indicated by arrows

BoSBP4 contained an open reading frame of 945 bp without introns, encoding a polypeptide of 314 aa, corresponding to a predicted molecular weight of 37 kDa protein and with an isoelectric point of 4.25 (Fig. 1). The nucleotide sequence of the BoSBP4 gene acquired in this study has been submitted to the GenBank database under the accession number MH429605.

**Fig. 2:**
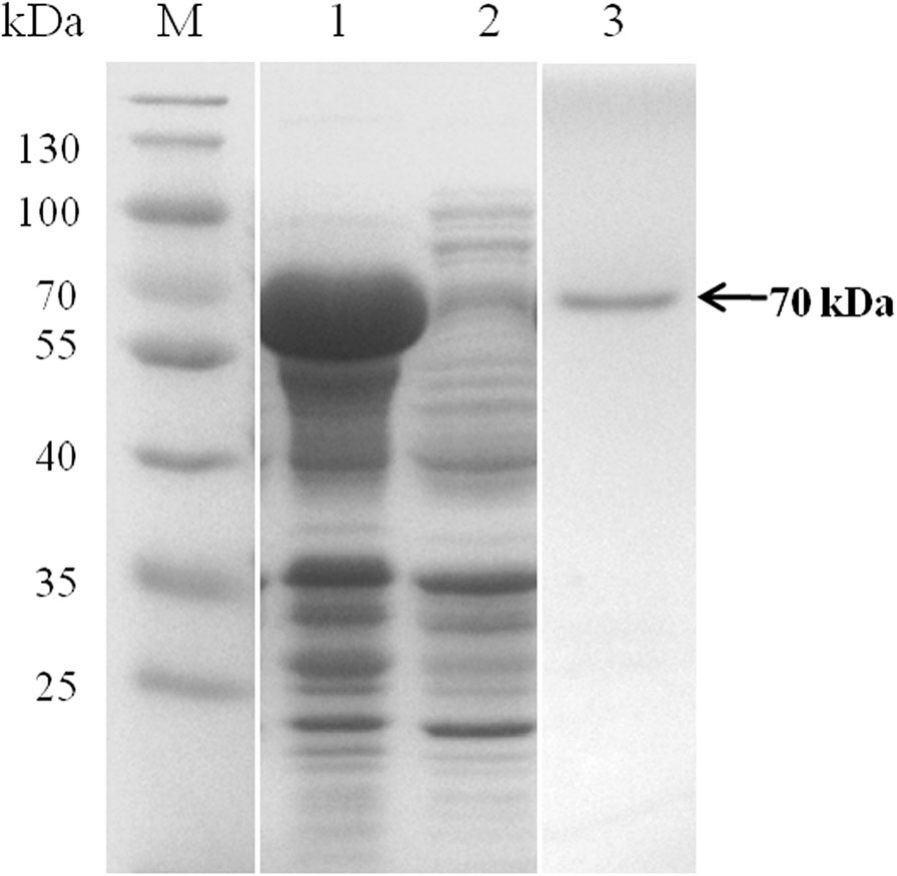
SDS-PAGE expression and purification of rBoSBP4. Lane M: molecular weight marker; Lane 1: lysate of IPTG induced pGEX-6P-1-BoSBP4; Lane 2: lysate of un-induced pGEX-6P-1-BoSBP4; Lane 3: purified product of rBoSBP4. The corresponding bands are indicated by arrows

As a signal peptide was cleaved between amino acids 22 and 23, and for the convenience of prokaryotic expression, BoSBP4 gene was truncated and expressed from 70 to 945 bp encoding 291 amino acids. The truncated BoSBP4 gene was expressed as GST-fusion protein in *E. coil* BL21 (DE3) with a predicted molecular mass of 61 kDa. However, the practical molecular weight of the rBoSBP4 in SDS-PAGE was about 70 kDa. Finally, the 70 kDa band in SDS-PAGE was identified with MALDI-TOF method, and the result corresponded to the SBP4 of *B. bovis*, probably due to a fold present in BoSBP4. The rBoSBP4 was extensively expressed in the supernatant and was then purified (Fig. 2).

### Phylogenetic and bioinformatics analyses

The amino acid sequence of BoSBP4 was blasted with the SBP4 sequences in the database. It was found that BoSBP4 showed a query cover of 89% and an identity of 40% with SBP4 of *B. bovis*, and the similarity was mainly located in the N terminus and the signal peptide site. Additionally, BoSBP4 shared 83% query cover and only 30% identity with SBP4 of *B. bigemina*. The amino acid sequence blast revealed that BoSBP4 was more similar to the SBP4 of *B. bovis* in structure. Amino acid sequence comparison of BoSBP4 with BoSBP3 revealed a query cover of 62% and an identity of 31%. The similarity mainly ranged from 185 to 290 aa, which may be a conserved domain in the SBP family.

**Fig. 3:**
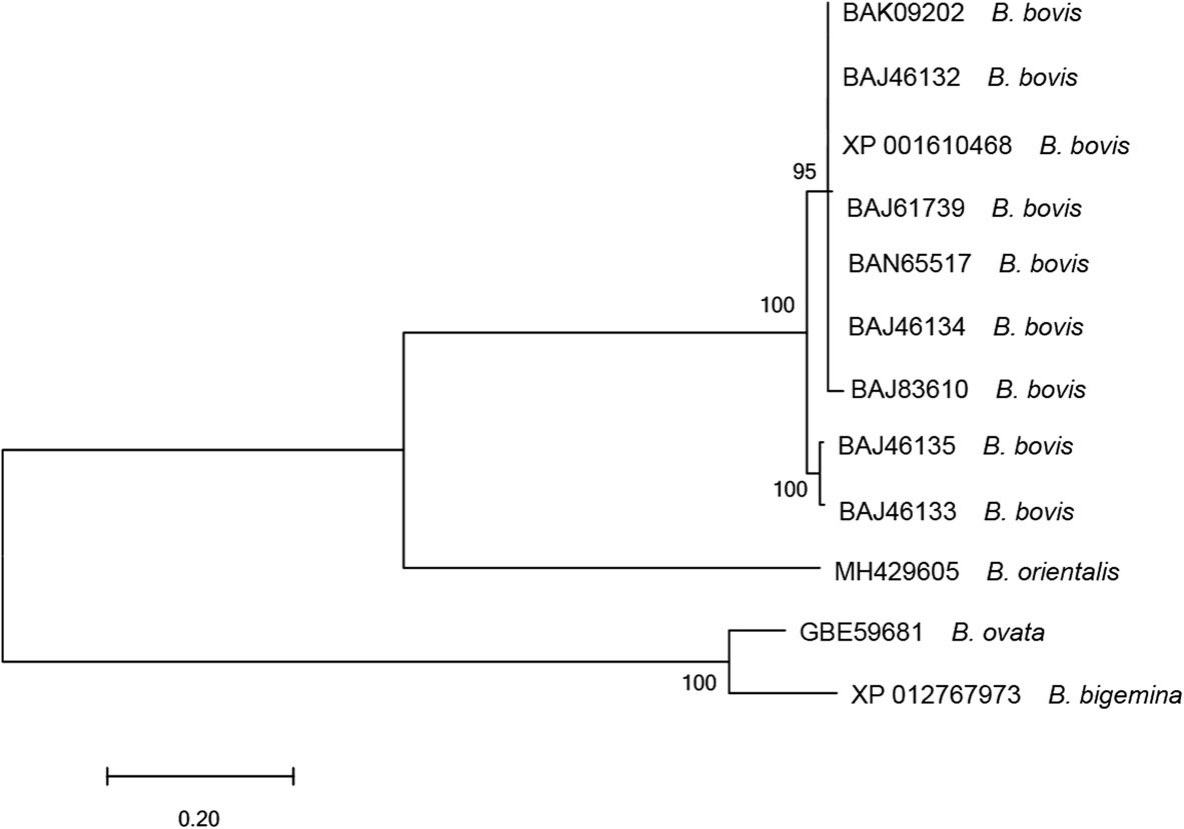
Molecular phylogenetic analysis of SBP4 amino acid sequences by the maximum likelihood method. The tree is drawn to scale, with branch lengths measured in the number of substitutions per site. The analysis involved 12 amino acid sequences. All positions containing gaps and missing data were eliminated. There were a total of 219 positions in the final dataset. GenBank accession numbers are indicated

The evolutionary relationships were analyzed according to the amino acid sequences of SBP4 from *B. bovis*, *B. bigemina*, *Babesia ovata* and *B. orientalis* (Fig. 3). The SBP4 proteins of *B. orientalis* and *B. bovis* were grouped in one clade, and those of *B. ovata* and *B. bigemina* were grouped in another clade. However, BoSBP4 and SBP4 of *B. ovata* and *B. bigemina* were grouped separately. The phylogenetic analysis indicated that BoSBP4 is more close to that of *B. bovis*, and the SBP4 of *B. ovata* is more close to that of *B. bigemina*. In contrast, BoSBP4 was more distantly related to that of *B. ovata* or *B. bigemina*.

The BoSBP4 gene was predicted to contain one exon and no intron and lack transmembrane regions and GPI anchor sites. The prediction results were consistent with the bioinformatics prediction of the exportome of *B. bovis*, no transmembrane regions or GPI sites but a signal peptide [43]. The motif scanning of the predicted amino acid sequence of BoSBP4 was performed. It consisted of a N-glycosylation site between 220 and 223 aa, seven casein kinase II phosphorylation sites in the range of 118 to 314 aa, four protein kinase C phosphorylation sites in the range of 5 to 212 aa, two tyrosine kinase phosphorylation sites, one glutamic acid-rich region profile (282–314 aa) and one FAINT region (171–249 aa).

Hydrophilicity, flexible regions, antigenic index and surface probability were predicted based on the corresponding algorithms, including Kyte-Doolittle, Karplus-Dchulz, Jameson-Wolf and Emini. There were far more hydrophilic regions than hydrophobic regions, with good hydrophilic regions accounting for approximately 75% of the total number of amino acid residues. Flexible properties were largely related with the amount of helix, turn and coil. The structures of α-helix and β-sheet were undeformable and hard to bind antibodies, which might compromise flexibility. However, turn and coil were similar to the protruding structures, which may facilitate binding with antibodies and serve as potential epitopes. High antigenic regions were uniformly distributed throughout the amino acid sequence and mostly concentrated in the range of 110 to190 aa. The main regions present on the protein surface might be located between 278 and 314 aa. Potential B cell epitopes can be estimated according to these four indices: good hydrophilicity, high flexibility, high accessibility and good antigenicity. Based on comprehensive analysis above, two regions were most likely to be potential B cell epitopes: 107–118 aa and 278–314 aa.

**Fig. 4:**
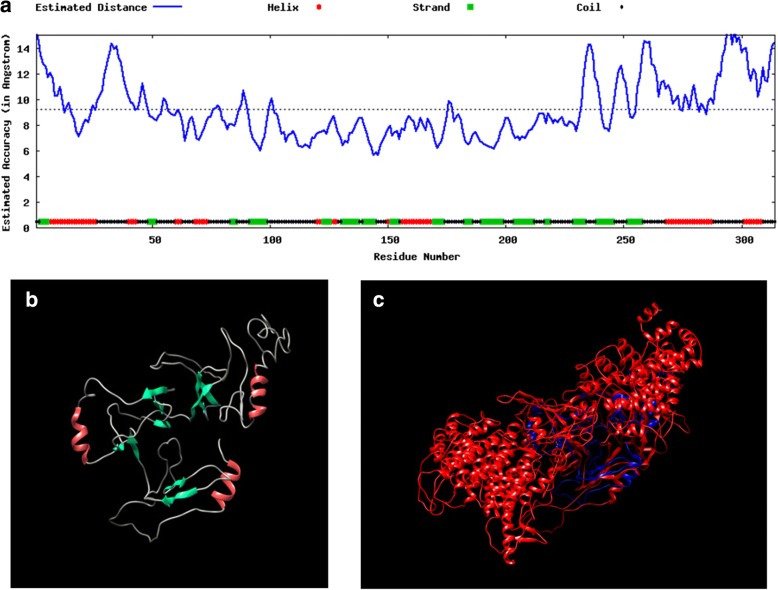
Schematic illustrations of predicted domains and crystal structures of SBP4. **a** The estimated local accuracy of the predicted structure of BoSBP4. **b** The predicted tertiary structure of BoSBP4 showing helix (red), strand (green) and coil (gray). **c** BoSBP4 structure is shown in blue, while the structural analog of APC is displayed in red

The three-dimensional structure of BoSBP4 was predicted with the I-TASSER software, with helix, strand and coil distributed throughout the BoSBP4. The schematic illustration of the predicted crystal structure of SBP4 is shown in Fig. 4. Ten proteins were selected due to their similarity in structure to BoSBP4, with the highest score for the anaphase-promoting complex (APC) protein. APC is a ubiquitin ligase that marks target cell cycle proteins for degradation by the 26S proteasome [44]. The functions of BoSBP4 were speculated to be most similar to those of the APC, because similar structures may lead to similar functions. The function of APC can serve as a reference for further research on BoSBP4.

### Identification of the immunoreactivity and native form of BoSBP4

**Fig. 5:**
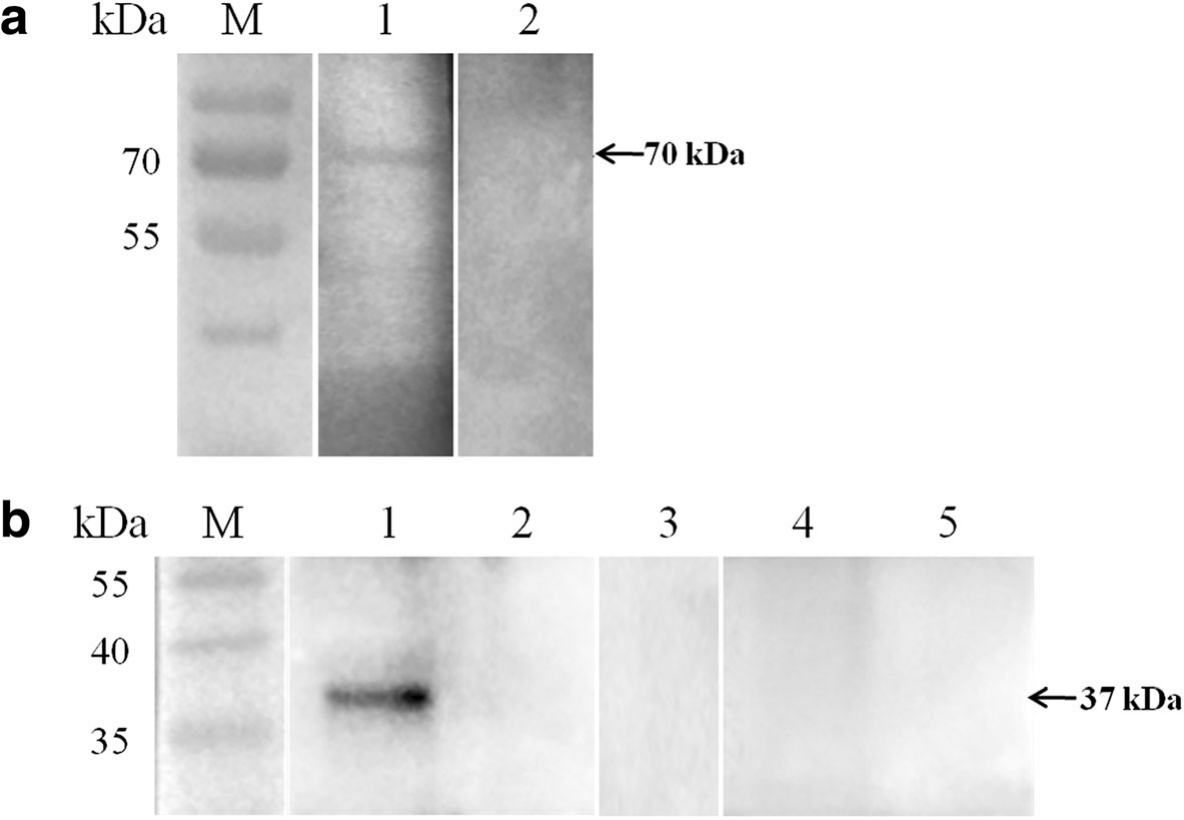
Western blot identification of immunoreactivity and native form of BoSBP4. **a** Lane M: molecular weight marker; Lane 1: rBoSBP4 probed with the serum of *B. orientalis*-infected buffalo; Lane 2: rBoSBP4 probed with the serum of uninfected buffalo. **b** Identification of the native form of BoSBP3 in *B. orientalis* merozoite lysate. Lane M: molecular weight marker; Lane 1: reaction of lysate of *B. orientalis*-infected erythrocytes with the serum against rBoSBP4; Lane 2: reaction of lysate of uninfected buffalo erythrocytes with the serum against rBoSBP4; Lane 3: lysate of *B. orientalis*-infected buffalo erythrocytes probed with anti-GST tag mouse monoclonal antibody; Lane 4: lysate of *B. orientalis*-infected buffalo erythrocytes probed with the serum of naïve mouse; Lane 5: lysate of uninfected buffalo erythrocytes probed with the serum of naïve mouse. The corresponding bands are indicated by arrows

The immunoreactivity of BoSBP4 was evaluated by incubating rBoSBP4 with the serum from *B. orientalis-*infected water buffalo. A band of about 70 kDa was detected, indicating the generation of antibodies against BoSBP4 in the *B. orientalis-*infected water buffalo (Fig. 5a). Meanwhile, no band was detected in the negative controls. Combined with the predicted secondary structure, the band revealed that BoSBP4 may be a good antigen for the detection of *B. orientalis*.

The expression of BoSBP4 in the merozoite was evaluated by incubation of the lysate of *B. orientalis-*infected erythrocytes with anti-rBoSBP4 serum. A specific band of about 37 kDa was detected, which was consistent with the expected molecular mass of BoSBP4 (Fig. 5b). However, no band was observed in negative controls. This experiment proved that BoSBP4 was actually present in the *B. orientalis* merozoite.

### Localization of BoSBP4

**Fig. 6:**
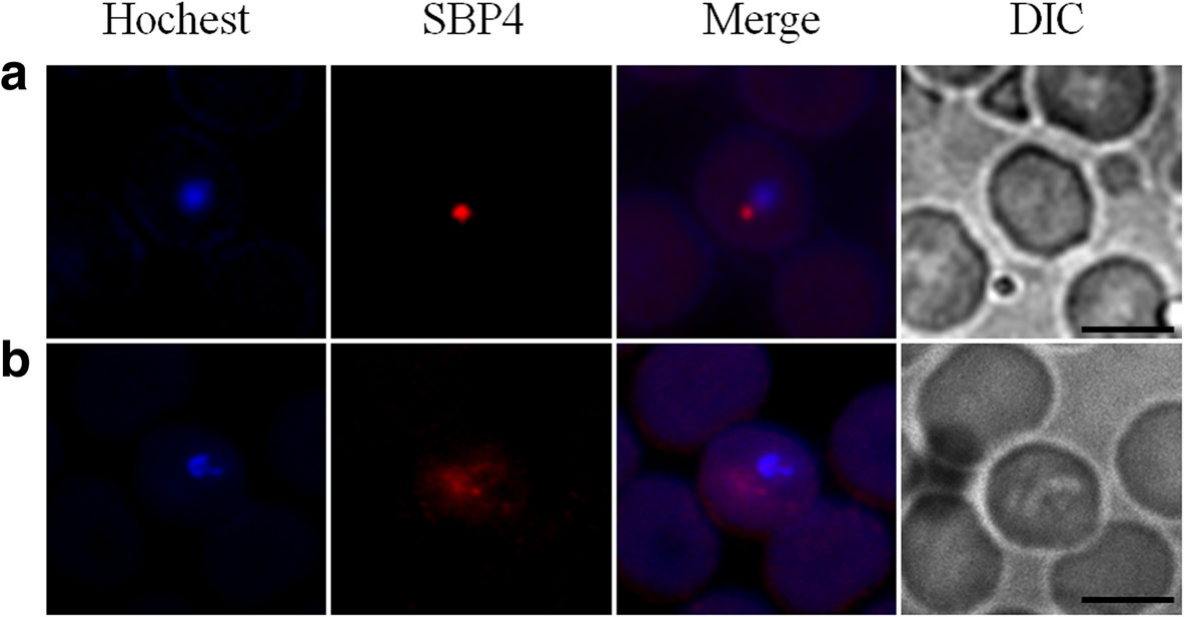
Localization of BoSBP4. Immunofluorescence and electron microscopy observation of BoSBP4. Anti-rBoSBP4 serum (red) and nucleus staining of Hoechst (blue). **a** During the early post-invasion stage, BoSBP4 was adjacent to the merozoite nucleus. **b** At the post-invasion stage, BoSBP4 was beginning to be secreted into the cytoplasm of iRBC. *Scale-bars*: 5 μm

For a better understanding of the properties of BoSBP4, IFA was applied to evaluate the localization of BoSBP4 in merozoite and iRBC. Fluorescence was detected in the intracellular phase. In the iRBC, BoSBP4 was located adjacent to the merozoite nucleus, followed by the diffusion of its fluorescence into the iRBC cytoplasm as puncta-like specks and a gradual increase of the fluorescence (Fig. 6). Meanwhile, no fluorescence could be observed in negative controls.

## Discussion

Due to few studies having been performed so far on *B. orientalis*, its mechanism and properties remain largely unknown. Recently, the mitochondrial and apicoplast genomes of *B. orientalis* were sequenced and analyzed as a supplement to genome analysis (unpublished data), which could facilitate a preliminarily understanding of *B. orientalis* [3, 30, 31]. *B. orientalis* is an obligate intracellular apicomplexan parasite sharing highly similar characteristics with *Plasmodium* spp. in many aspects [8, 26]. For *Plasmodium* spp., the first crucial step is to invade the host cell using several proteins secreted by apical organelle complex. For example, apical membrane antigen 1 (AMA1) secreted by micronemes was reported to achieve tight junction formation between parasite and host cell by combining with rhoptry neck proteins [10–12]. The AMA1 of *B. orientalis* was merely identified and characterized in the merozoite [45], and further research on the AMA1 of *B. orientalis* could refer to the AMA1 in *Plasmodium* and *Toxoplasma gondii* that had been extensively studied in molecular functions and related mechanisms [46, 47]. On the other hand, many differences existed in related proteins and mechanisms between *Babesia* spp. and *Plasmodium* spp. For *Plasmodium* spp., there were four entirely different stages involved in the whole process in the iRBC, including ring stage (0–24 h), trophozoite stage (24–36 h), schizogony stage (36–48 h) and the final merozoite stage [48–50]. However, in *Babesia* spp., merozoite was the only stage found in the iRBC, leading to far less time in iRBC for *Babesia* (approximately 6–8 h) than *Plasmodium* spp. (approximately 36–72 h) [8, 51]. Therefore, the molecular mediated parasites residing in the host cell might be fewer and more infertile for *Babesia* than *Plasmodium* spp. In *Plasmodium* spp., many proteins were reported to be discharged into the cytoplasm or cytoplasmic face of iRBC to internalize and stabilize the host cell environment for parasite survival and replication [16–18, 52–54]. For example, ring-infected erythrocyte surface antigen (RESA) proteins were secreted by dense granules, and then were located to the cytoplasmic face of iRBC through binding the repeat 16 of beta-chain for stabilizing the spectrin tetramer against mechanical and thermal damage [16, 17, 54]. Dense granule antigens (GRAs), secreted by *Toxoplasma gondii*, were regarded as important targets for the diagnosis and development of vaccines [55, 56].

However, there is no consensus sequence in *Babesia* spp. Instead of dense granules, the spherical body is a unique organelle present in the apical organelle complex of *Babesia* spp. [19]. So far, four SBPs (SBP1, SBP2, SBP3 and SBP4) have been described for *B. bovis* in several articles, and for *B. bigemina*, only the nucleotide sequences of SBP3 and SBP4 were submitted to NCBI [19–21, 24]. Recently, a study has identified for the first time the presence of SBP3 in *B. orientalis*, which was observed to be secreted into the cytoplasm of iRBC [26].

In this study, the SBP4 gene in *B. orientalis* was firstly cloned and sequenced, which had an open reading frame of 945 bp with no intron. Meanwhile, BoSBP4 was truncated and expressed for the convenience of prokaryotic expression, because there was a predicted signal peptide cleave site between 22 and 23 aa. For a better understanding of the genetic relationship of SBP4 among different species, the amino acid sequences were blasted with each other. The amino acid homology of BoSBP4 to SBP4 of *B. bovis* and *B. bigemina* was 40% and 30%, respectively, indicating that BoSBP4 was more closely related to *B. bovis*, which is consistent with the phylogenetic analysis between *B. orientalis* and *B. bovis* in *18S* rRNA, AMA1, heat shock protein 90 (HSP90) and HSP70 sequences [3, 45, 57, 58]. BoSBP4 shared 31% identity with BoSBP3 in the range of 185 to 290 aa, demonstrating a conserved region in the SBP family. For the prediction of motifs in BoSBP4, the phosphorylation sites throughout the BoSBP4 may indicate the extensive modifications in BoSBP4. BoSBP4 also contained one FAINT region from 171 to 249 aa and the motifs of BoSBP4 shared high similarity in structure with SBP4 of *B. bovis*, implying the similarity in essential functions.

Furthermore, in IFA, the fluorescence of BoSBP4 was observed in the intracellular process. Here, BoSBP4 was discharged into the cytoplasm of iRBC as puncta-like specks and gradually increased. The secretion of BoSBP4 into the host cell may be related to the presence of a signal peptide in the N-terminus as a guide. So far, there has been no report about the secretion of other proteins into the iRBC except for SBPs at the late stage of invasion in *Babesia* spp., suggesting the significant roles of SBPs in the intracellular period. Furthermore, SBPs were identified to be secreted into different regions. SBP1 and SBP2 in *B. bovis* were characterized to be discharged and concentrated near the inside of the erythrocyte membrane, while SBP3 and SBP4 in *B. bovis* and BoSBP3 were identified to be secreted throughout the cytoplasm. Moreover, the degree of secretion might vary in SBPs. BoSBP4 was slightly less than BoSBP3 in the fluorescence when discharged into the cytoplasm.

Despite extensive research on SBPs, their mechanisms and functions remain to be further explored [19, 22, 23]. Based on the research of *Plasmodium* spp., some hypotheses are proposed for *Babesia* spp. In *Plasmodium* spp., parasitophorous vacuole (PV) will remain until merozoite egression from the iRBC [59–61]. Additionally, the PV membrane (PVM) is considered to isolate the parasites from the cytoplasm of iRBC to maintain a stable microenvironment (a neutral PH) and act as an intermediate between parasites and RBCs [61]. Furthermore, dense granule proteins were reported to participate in host cell internalization [62]. However, for *Babesia* spp., PVM will disappear with its invasion into the host cell [4]. The special process for *Babesia* spp. is proposed to be associated with the unique organelle, spherical body, and this needs to be further investigated. Another hypothesis about *Babesia* spp. is that SBPs are related to the formation of ridges. After invasion into the host cell, parasites will modify RBC in morphology, physiology and biochemistry [53, 63–65]. Some ridge-like protrusion “ridges” will be present in the whole RBC membrane surface infected by *Babesia* spp. [63]. They are similar to the “knobs” present in *Plasmodium*-infected RBC, and these “knobs” consist of some parasite-derived proteins, such as *P. falciparum* erythrocyte protein 3 (PfEMP3) and KAHRP [53, 63]. For *Babesia* spp., merely spherical body proteins are known to be released into the iRBC at the late stages. Therefore, SBPs are considered to be related with the formation of “ridges”. In addition, the parasitized RBC will adhere to some other cells for immune escape, such as endothelial cells, uninfected RBC and dendritic cells [64, 66]. The reported related parasite-derived and adhesive protein in *P. falciparum* is *P. falciparum* erythrocyte protein 1 (PfEMP1) that is secreted into the surface of iRBC [52]. In *Babesia* spp., SBP1 and SBP2 were characterized to localize to the erythrocyte membrane, which may play the same vital roles in adhesion [19, 22]. The aforementioned assumptions about *Babesia* spp. should be further tested to facilitate a better understanding of the mechanism and functions of SBPs.

Even though the invasion stage is vital to parasites, they will be exposed to the host immune system for a far longer time and with more risks at the post-invasion stage than at the invasion stage. Therefore, the mechanism for the post-invasion stage is worthy of further investigation.

## Conclusions

In this study, a novel spherical body protein in *B. orientalis* was identified to be secreted into the cytoplasm during its residence in the erythrocyte. BoSBP4 might be functionally important in interaction between the parasite and the host cell to facilitate the parasite survival and multiplication in the erythrocyte. Furthermore, BoSBP4 can be clearly recognized in the serum of *B. orientalis-*infected water buffalo, suggesting the potential application of BoSBP4 to *B. orientalis* diagnosis. The present study will facilitate the understanding of the SBPs and the development of an effective therapy and prevention against *B. orientalis* infection.

## Abbreviations

AA: Amino acid
AMA-1: Apical membrane antigen-1
BoSBP4: The SBP4 in *Babesia orientalis*
cDNA: Complementary DNA
CDS: Sequence coding for amino acids
ELISA: Enzyme-linked immunosorbent assay
gDNA: Genomic DNA
GPI: Glycosylphosphatidy-l-inositol
IFA: Indirect immunofluorescence assay
IP: Isoelectric point
IPTG: Isopropyl-β-D-thiogalactopyranoside
iRBC: Infected red blood cell
LOMETS: Local meta threading server
ORF: Open reading frame
PBS: Phosphate-buffered saline
PBST: Phosphate-buffered solution Tween-20
PMSF: Phenylmethylsulfonyl fluoride
PV: Parasitophorous vacuole
rBoSBP4: Recombinant BoSBP4
RESA: Ring-infected erythrocyte surface antigen
SBPs: Spherical body proteins
SDS-PAGE: Sodium dodecyl sulfate polyacrylamide gel electrophoresis
TBST: Tris-buffered saline Tween-20
TEN: Tris/EDTA/NaCl
TM: Transmembrane
